# The Effect of Organic and Mineral Fertilizers on Silage Maize Biomass Yield and Quality Across Different Soil–Climate Conditions in the Czech Republic

**DOI:** 10.3390/plants15081231

**Published:** 2026-04-16

**Authors:** Lukáš Hlisnikovský, Ladislav Menšík, Muhammad Roman, Jaffar Iqbal, Veronika Zemanová, David Kincl, Pavel Nerušil

**Affiliations:** 1 Department of Nutrition Management, Czech Agrifood Research Center, Prague–Ruzyně, Drnovská 507, 161 00 Prague, Czech Republic; ladislav.mensik@carc.cz (L.M.); m.maan26@outlook.com (M.R.); jaffariqbal987@gmail.com (J.I.); veronika.zemanova@carc.cz (V.Z.); pavel.nerusil@carc.cz (P.N.); 2 Department of Environment, Faculty of Environment, Jan Evangelista Purkyně University, Pasteurova 15, 400 96 Ústí and Labem, Czech Republic; 3 Department of Pedology and Soil Conservation, Research Institute for Soil and Water Conservation, Žabovřeská 250, 156 00 Prague, Czech Republic; kincl.david@vumop.cz

**Keywords:** *Zea mays* L., farmyard manure, mineral fertilizers, soil–climate conditions, soil carbon, feedstuff quality

## Abstract

Maize biomass production and quality are influenced by numerous factors, including fertilization, soil characteristics, and climatic conditions. The aim of our study was to evaluate how different fertilization treatments ((1) Control, (2) farmyard manure (FYM), (3) FYM with added mineral nitrogen (FYM + N), and (4) FYM with added NPK mineral fertilizers (FYM + NPK)) affect the biomass yield and quality parameters (crude protein (CP), fiber content (FC), neutral detergent fiber (NDF), starch content (STR), organic matter digestibility (OMD), and neutral detergent fiber digestibility (DNDF)) of silage maize under various soil and climatic conditions in the Czech Republic (Caslav—degraded Chernozem, Ivanovice na Hané–Chernozem, Lukavec–Cambisol). The experiment was conducted from 2020 to 2023. Additionally, the study analyzed the effects of fertilization on soil chemical properties (pH, P, K, Ca, Mg, C, N). The highest average biomass yields were recorded in Ivanovice (23.8 t ha^−1^, A), followed by Lukavec (19.7 t ha^−1^, B) and Caslav (18.1 t ha^−1^, B). Comparing fertilizer treatments, no significant differences were observed among FYM, FYM + N, and FYM + NPK; however, all three treatments significantly outperformed the Control at all sites. Conversely, fertilization did not affect the quality parameters. For silage maize, FYM represents the optimal fertilization strategy, providing yields and quality comparable to the combined application of mineral N, P, and K, which are more costly (in terms of purchase and application) and, under certain conditions, may negatively impact the environment. Nevertheless, the application of mineral fertilizers increased soil nutrient content, thereby improving conditions for subsequent crops.

## 1. Introduction

Maize is one of the three most important crops grown worldwide (following wheat and rice) and is used and utilized in many industries, such as the food and distillery industries [1,2,3,4], as a basic raw material for livestock feed [5], and in the energy [6,7] and cosmetics industries [8].

Maize is grown for grain or as a feedstock for silage production. In the Czech Republic, the latter option predominates, with grain maize being grown on approximately 84,000 ha, while silage maize covers an area of approximately 227,000 ha (2015–2022) [9]. With such a large area, it is the fourth most widely grown crop in the Czech Republic, following wheat, winter rape, and barley.

One of the key questions in the cultivation of silage maize is how to fertilize it properly in order to achieve optimal results. Compared to other cereals, silage maize produces a great amount of biomass. This process requires a high intake of nutrients, mainly nitrogen (N). On the other hand, maize is more efficient at N utilization in comparison with other cereals [10]. The high requirement of nutrients is usually compensated for with mineral fertilizers, organic manures, or with residues of preceding crops [11,12,13,14,15,16,17]. All types of fertilizers (manures, organic, and mineral) have their pros and cons. Mineral fertilizers have a precisely specified composition, allowing accurate dosing, and are quickly available to crops. But they are costly, and the price strongly depends on the geopolitical situation [18,19], and can negatively impact the environment and human and animal health [20,21,22]. Manures represent a by-product of animal husbandry and are readily available to farmers who engage in animal production. However, they are not homogeneous like mineral fertilizers; their composition varies depending on many variables (season, stage of development, and species of animal, diet, housing technology, etc.). The ability of manures to release nutrients into the environment depends heavily on the C:N ratio. Fertilizers with a low C:N ratio (slurries) usually mineralize very quickly, with most nutrients available in the first year of application, while high C:N ratio manures (farmyard manure) mineralize slowly, releasing their nutrients for a longer period in lower amounts [23,24,25]. However, the greatest advantage of manures is the presence of available carbon (C), which is a fundamental element in maintaining and increasing soil fertility and biodiversity [26,27,28,29].

Another of several factors affecting maize biomass production is the weather. Maize is a C4 plant, a warm-season crop, requiring specific soil–climatic conditions for optimal performance, such as areas with moderate to high temperatures, well-distributed precipitation, and without frosts, to which it is very sensitive. Excessive moisture and prolonged droughts can cause a yield and quality reduction, especially during critical stages (flowering, grain development), making the maize vulnerable to climate change [30]. Such optimal soil–climate conditions can be found in the South Moravian region in the Czech Republic. However, maize is admired among farmers even in less favorable areas characterized by higher latitudes, colder climate with higher precipitation, and less fertile soils like Cambisols. Climate change affects all areas of our planet, and the Czech Republic is no exception. Climate change manifests itself in rising temperatures [31], varying precipitation patterns [32], and more frequent occurrences of extreme droughts [33,34], generally connected with negative impacts on crop production [35]. In the Czech Republic, these factors will have a negative influence primarily on the lowlands in the South Moravian region. According to Maitah et al. [36], the average maize yield decreased in the Czech Republic after 2010 due to reasons related to climate change, and can be expected in Germany in the near future, too [37]. However, some studies do not only forecast negative impacts of climate change on plant production, but also the expansion of new crops, not traditionally grown before, into conditions that are or will be altered because of global warming. Better conditions for certain crops can be expected in the uplands in the Czech Republic [38], including maize [39], and this prediction of better performance of maize in northern and higher areas can be assumed in the whole of Europe [40,41].

To determine how silage maize hybrid Prestol will respond to different fertilizer treatments and various soil and climatic conditions in the Czech Republic, we evaluated a four-year period of cultivation (2020–2023) in this article. The trials were conducted at three locations characterized by different soil–climatic conditions: (1) Caslav (degraded Chernozem); (2) Ivanovice na Hane (Chernozem); and (3) Lukavec (Cambisol). The aim of this research was to evaluate the response of maize to four fertilizer treatments, including (1) Control (unfertilized); (2) cattle farmyard manure (FYM); (3) FYM with addition of mineral N (FYM + N); and (4) FYM with addition of mineral NPK (FYM + NPK). Specifically, we analyzed the effect of fertilization and location on the yield and quality parameters of biomass: (1) crude protein (CP); (2) fiber content (FB); (3) neutral detergent fiber (NDF); (4) starch content (STR); (5) organic matter digestibility (OMD); and (6) digestible neutral detergent fiber (DNDF). The assessment also includes an evaluation of the impact of fertilization on soil properties (pH; phosphorus–P; potassium–K; magnesium–Mg; calcium–Ca; total carbon–C; and total nitrogen–N) and analysis of climate conditions.

## 2. Results

### 2.1. Effect of Fertilizer Treatments on Maize Biomass Yield (BIY) and Soil Chemical Properties

The complete results of the effect of fertilizer treatments on the dry matter biomass yield (BIY) of silage maize at various locations are presented in Table 1.

Soil and climatic conditions represent fundamental natural factors that affect the biomass productivity of silage maize. According to the multivariate analysis of variance (MANOVA) results, the BIY was mainly affected by locality (49%; *p* < 0.05), followed by fertilizer treatment (27%; *p* < 0.05) and season (24%; *p* < 0.05). The most favorable conditions for biomass production were found in Ivanovice, where the average BIY across all years and fertilization treatments was highest (23.8 ± 0.8 t ha^−1^, B). In contrast, the average yields in Caslav (18.1 ± 0.3 t ha^−1^, A) and Lukavec (19.7 ± 0.6 t ha^−1^, A) were significantly lower, and statistically comparable to one another (the letters, following the means, represent significant differences between areas). In Ivanovice, the weather conditions during each year of the trial were the primary factors (87%) influencing BIY, while fertilization had a negligible effect (13%). In Caslav and Lukavec, however, the significance of both factors was reversed, with fertilization affecting the BIY by 67% and 61%, respectively, while the effect of seasons was lower.

The effects of the individual fertilizer treatments were consistent across all sites. The Controls yielded the lowest BIY. In contrast, all other treatments (FYM, FYM + N, FYM + NPK) produced significantly higher yields. Additionally, BIYs were comparable among the fertilizer treatments throughout the entire trial period (Table 1; the most upright column). In terms of BIY, the most significant finding is that FYM applied alone provided sufficiently rich nutrition for silage maize, resulting in BIY comparable to those achieved with the FYM + N and FYM + NPK treatments. The addition of mineral fertilizers (NPK) led to yield increases of 7%, 8%, and 13% in Ivanovice, Caslav, and Lukavec, respectively.

**Figure 1:**
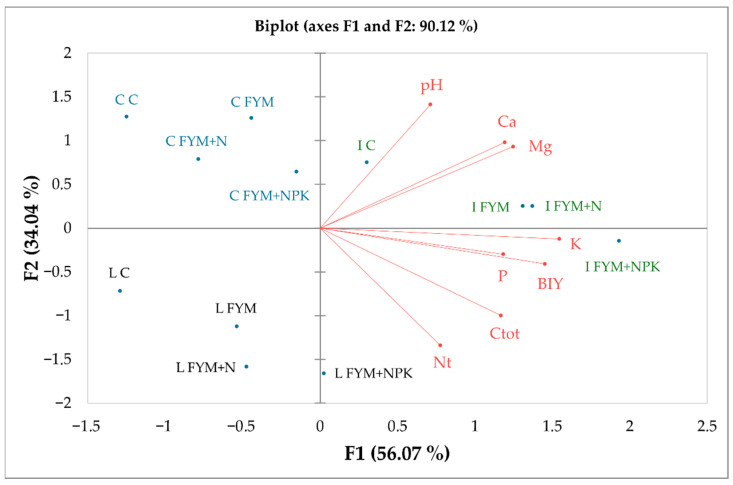
Result of the PCA showing relationships between localities, fertilizer treatments, soil chemical parameters, and BIY. Abbr. of localities: C—Caslav (blue); I—Ivanovice (green); L—Lukavec (black). Abbr. of fertilizer treatments: C—Control (unfertilized); FYM—farmyard manure; FYM + N—farmyard manure with mineral N; FYM + NPK—farmyard manure with mineral NPK. Soil chemical elements and the maize biomass yield (BIY) are colored red.

The soil in Ivanovice (Chernozem) is naturally fertile; even the unfertilized Control exceeds the fertility levels of the fertilized treatments from the Caslav and Lukavec (Table 2, Figure 1).

The application of FYM and mineral nutrients further enhances soil fertility in Ivanovice, beneficially influencing the nutrient content in the soil (Table 2, Figure 1), which is positively correlated with the BIY (Figure 1). However, maize could fully benefit from the high fertility only under particularly favorable climatic conditions, as observed in 2022 (Table 4). This year was characterized as very warm, with a normal level of precipitation (Table 4), which suits C4 plants very well. In Caslav and Lukavec, the seasons were mostly evaluated as “very warm” or “extraordinarily warm” (Table 4), usually with normal precipitation. The only exception was found in Lukavec, where the year 2020 was characterized as “very wet” and “extraordinarily warm”. This combination caused the lowest BIY in this area (Table 1) during the evaluated period.

The chemical analysis of soil parameters revealed differences between localities, confirming the exceptional fertility of Chernozem in Ivanovice, and separating all three localities from each other (Figure 1), forming three various clusters. Chernozem in Ivanovice is naturally a highly fertile soil, providing a sustainable amount of nutrients for crop production even in the unfertilized Control treatment. In Figure 1, we can see that the “I C” (Ivanovice Control treatment) is found close to FYM + NPK treatments from Caslav and Lukavec, which are the most fertile soils in the analyzed areas. Application of FYM and FYM + NPK fertilizers even enhances the soil fertility in Ivanovice, far beyond the possibilities of the soils in Caslav and Lukavec.

The soil in Caslav exhibits similar characteristics to that of Lukavec (Table 2, Figure 1). However, the soil has lower soil C and N content, which are parameters strongly correlated with BIY (Figure 1). According to the PCA, the unfertilized Control treatment in Lukavec is the closest treatment to those from Caslav. Conversely, when the Lukavec soil is treated with FYM and mineral fertilizers, its properties become more like those of the Ivanovice soils. Thus, the soil in Lukavec represents a transitional inter-cluster between the soils in Caslav and Ivanovice (Figure 1), shifting towards one side or the other depending on the fertilization method employed. Therefore, the application of fertilizers in the areas of Caslav and Lukavec plays a crucial role, as fertilizers compensate for the natural fertility of Chernozems.

The combined application of FYM + NPK significantly increased N, P, K, and C contents across all localities. The FYM, applied alone, enhanced C and N levels; however, it has limited effects on P and K unless combined with inorganic fertilizers (Table 2).

In terms of fertility, soil type represents a natural barrier that can be influenced to some extent by human activity, such as fertilization. The application of mineral fertilizers (NPK) can improve soil fertility by increasing the availability of individual nutrients. However, even well-fertilized, less fertile soils may not achieve the natural fertility of Chernozem (Ivanovice na Hané).

### 2.2. Silage Maize Quality Parameters

In contrast to the BIY, the qualitative parameters (CP, FB, NDF, STR, OMD, and DNDF) were not affected by locality or fertilizer treatment. According to the MANOVA and ANOVA results, no significant differences between the factors were found. The complete results are shown in Table 3.

**Figure 2:**
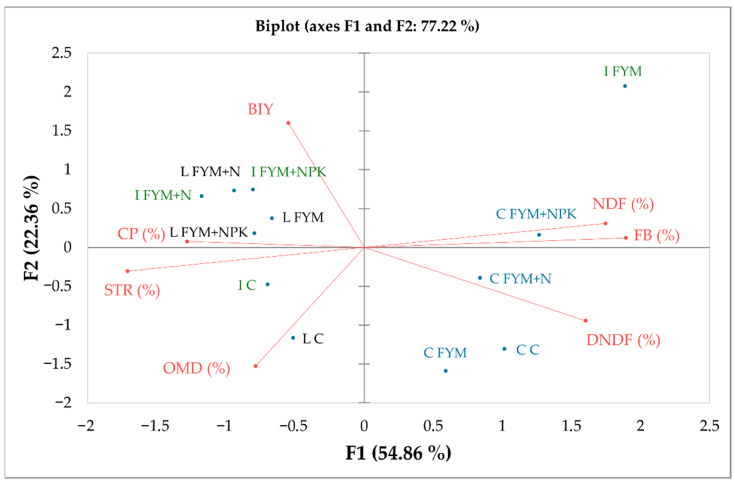
Result of the PCA showing relationships between localities, fertilizer treatments, maize quality parameters, and BIY. Abbr. of localities: C—Caslav (blue); I—Ivanovice (green); L—Lukavec (black). Abbr. of fertilizer treatments: C—Control (unfertilized); FYM—farmyard manure; FYM + N—farmyard manure with mineral N; FYM + NPK—farmyard manure with mineral NPK. Maize quality parameters and the maize biomass yield (BIY) are colored red.

Individual fertilization treatments and different soil–climatic conditions thus provided biomass of comparable quality. Although the differences in qualitative parameters were not statistically significant, the PCA (Figure 2) was able to divide the individual locations and fertilizer treatments.

In the graph of component weights F1 and F2, the first two axes are significant, together representing approximately 77% of the variability (Figure 2: biplot, yields, and forage quality). In the F1 vs. F2 graph, the F1 axis clearly characterizes the FB, NDF, and DNDF parameters (r = 0.82 to 0.97, positive correlation) and the STR parameter (r = −0.88, negative correlation). On the PC2 axis, a correlation was recorded with the BIY parameter (r = 0.82, positive correlation), as well as with OMD (r = −0.78, negative correlation).

In the scatter diagram of component scores (Figure 2: biplot, yields, and forage quality), locations and fertilization treatments (C, FYM, FYM + N, and FYM + NPK) are clearly divided along the F1 axis according to BIY, NDF, and DNDF content, and STR. Along the F2 axis, the locations and fertilization treatments are differentiated according to BIY. The Caslav (C) location differs significantly from the Lukavec (L) and Ivanovice (I) locations. This is due to its higher FB, NDF, and DNDF content and lower STR content, which may be caused by different soil conditions (lower-quality sandy loam soil) and lower water availability in the soil profile (frequent droughts, etc.). The FYM treatment (Ivanovice site) also differed significantly, having the second highest BIY yield, the highest FB and NDF content, but also the second lowest STR content and the lowest CP content (when starch is stored in above-ground biomass and grain, less N is available in the soil due to the combination of high mass yield and the fact that N is required for starch synthesis). At the Lukavec (L) and Ivanovice (I) sites, the Controls (C) differed significantly from the fertilized variants (the fertilized treatments are similar in terms of yield and forage quality parameters and are located close to each other in the cluster analysis). At the Caslav site, the Control and FYM treatments have similar parameters and differ further from the fertilized treatments (FYM + N and FYM + NPK).

## 3. Discussion

Extensive use of chemical fertilizers in intensive cropping systems has undoubtedly contributed to increased agricultural productivity in the past and nowadays. The availability of mineral fertilizers, especially N, together with new discoveries in genetics and plant breeding, agricultural technologies, and pesticides, led to unprecedented worldwide increases and booms in crop production after World War II [42]. However, after several decades of intensive use of mineral fertilizers and agricultural chemicals, the downside of this approach to plant production has been discovered and confirmed. Such disruptions in agroecosystems include soil degradation processes, like salinization, acidification, nutrient depletion, compaction, soil organic carbon decline, soil and water contamination, and biodiversity loss [43,44]. To address these issues, several sustainable approaches have been developed, such as conservation tillage, integration of cover crops, or application of organic amendments, such as farmyard manure (FYM). Manure is a fundamental element in plant nutrition and soil care. Due to mineralization, it enriches the soil with nutrients available to plants (reduced reliance on mineral fertilizers) and positively affects soil properties, such as microbial biomass, activity of soil enzymes, occurrence of biota, increased pH, and soil physical properties [45,46]. The effect of manure on soil and crops is variable, depending on soil type, rate and timing of application, and site-specific climatic conditions; therefore, all positive effects cannot be considered as a rule [47]. In our case, the FYM positively influenced the BIY at all three trial localities (Table 1). The lowest increase compared to the unfertilized Control was recorded in Ivanovice (17%), followed by Caslav (18%) and Lukavec (35%). Consistent with our findings, Yang et al. [48] also observed a 65% yield increase in wheat–maize rotation systems with FYM applied alone, reinforcing the idea that organic sources can sufficiently meet crop nutrient needs under certain conditions. Similar trends were also observed where cattle slurry significantly enhanced maize biomass production by 51–194% over the unfertilized Control [49,50]. These findings support the benefits of organic amendments, including their residual effects, which improve nutrient availability over time [51]. The FYM thus exhibited higher efficiency in a less fertile soil type (Lukavec–Cambisol). The fertile soils (Chernozems) already contain high levels of essential nutrients, and subsequent additions do not boost biomass yield because plants are already saturated. On the other hand, less fertile soil types contain low nutrient reserves, so the addition of readily available nutrients directly boosts plant growth and enhances the soil nutrient pool. The combined application of FYM and mineral fertilizers can significantly increase maize biomass yield when compared to Control and NPK treatments [52,53], but the positive effect depends on the soil and climatic conditions of a specific area, and it does not apply as a rule [54]. In our experiment, additional application of mineral forms of nutrients (N; NPK) resulted in slightly higher BIY (Table 1), but the differences were insignificant in comparison with the FYM treatment. Therefore, in the context of the Czech Republic, the added cost of mineral fertilizers may not be justified, given the marginal yield improvements when FYM is already in use. Additional NPK may be unnecessary without significant agronomic benefit, particularly for small- to medium-scale farmers. The results indicate that FYM alone can sustain silage maize production under Czech soil–climate conditions. Integrating organic sources into fertilization strategies enhances soil health and can reduce reliance on costly mineral fertilizers, contributing to more sustainable and environmentally friendly agricultural systems.

The addition of NPK was not related to significantly higher BIY. Maize was therefore not limited by a lack of nutrients, but by natural barriers represented by soil and climate conditions. Lower yields in Caslav and Lukavec (compared to Ivanovice) are the result of soil and climate barriers. In the case of Caslav, soil appears to be the limiting factor, while in Lukavec, it is both soil and climate. Maize is a C4 crop, preferring moderate to high temperatures, no frost periods, and well-distributed precipitation. Lukavec is located at higher altitudes with cooler weather. This area is more suitable for C3 plants, and traditionally, root crops are mainly grown here. The lowest BIY was recorded here in 2020 (Table 1), a year with an “extraordinarily wet” June, which could negatively affect biomass production by nutrients runoff and reduced nutrient availability. On the other hand, the highest BIY was recorded in Ivanovice in 2022 (Table 1). This year was characterized as “very warm”, with “normal” precipitation (Table 4), probably the optimal conditions for maize biomass production in this area. Our results point to a relationship between weather conditions and silage biomass production, but also to the ability of the Prestol hybrid to deliver high biomass yields under optimal conditions.

As mentioned above, the application of all types of fertilizers can significantly alter the chemical properties of the soil. In our study, the soil parameters were significantly affected by different fertilization amendments at all localities (Table 2, Figure 1). The higher soil pH was noted under FYM treatment, compared to other fertilization treatments, but the differences were insignificant. Application of mineral forms of P and K resulted in the highest concentration of these elements in the soil (Table 2), showing a beneficial effect of mineral fertilizers for restoring the pool of P and K, and ensuring a sufficient dose of critical elements for the following crops. The long-term omission of important macronutrients in the form of fertilization causes a decline in the concentration in the soil over time due to crop removal, weathering, and leaching, unless organic matter is continuously supplied and retained within the soil system [55,56]. Manures, organic fertilizers, and crop residues can partially replenish important nutrients via mineralization, but a complete lack of elements applied by fertilizers will ultimately lead to depletion. This is particularly problematic for plant production in the Czech Republic, which is heavily dependent on the application of mineral N, while the amount of P, K, and Ca fertilizers applied is at the same level as at the end of the 1950s [54]. The same crisis also affects all forms of manure, which is linked to a decline in livestock numbers and thus reduced availability of manure and slurry [54,57]. Dependence on mineral N and insufficient fertilization with other elements exacerbates the problem of soil degradation [58], especially acidification. One possible way to prevent this is to apply mineral fertilizers and organic manures together, representing a way recommended for sustainable and ecologically safe crop production [56,59,60]. This synergistic effect of integrated organic and inorganic fertilization initiates a balanced nutrient delivery system that supports both immediate plant needs and soil health improvements.

The study found that the fertilization treatment had a negligible effect on the quality parameters of silage maize biomass (Table 3). However, when it came to specific locations (soil–climate conditions), the PCA revealed differences.

According to the PCA, the comparison of three different localities with different soil and climatic conditions revealed differences in forage quality attributes. Previous studies showed that mineral fertilizers are crucial for enhancing both crop yields and quality characteristics, including protein, lipid, and carbohydrate contents [61,62]. Good-quality forage is typically characterized by NDF values below 50% of dry matter, which is also consistent with our findings and meets the criteria of good-quality forage as reported in the literature [63,64,65]. This criterion serves as an important benchmark for feed quality evaluation. The NDF values in this study ranged from 41.9% to 51.0%, remaining close to the threshold for high-quality forage (50%) [64]. However, the values observed were generally higher than those reported by Ferratto and Shaver [66], particularly at Ivanovice, where NDF reached 51.0% under FYM. In contrast, Lukavec consistently exhibited lower NDF values (41.9–44.5%). These differences indicate that site-specific climatic conditions, such as temperature and water availability, likely promoted greater structural fiber accumulation at Ivanovice compared to Lukavec. Previous studies, such as Nazli et al., 2016 [67], reported non-significant effects of cattle manure and inorganic fertilizers on fiber fractions in silage maize. Similarly, Ali et al., 2019 [64] reported no significant influence of inorganic and organic phosphorus-based fertilizer amendments on NDF. In agreement with these findings, the present study also showed no significant differences in NDF (41.9–51.0%) among fertilization treatments, including FYM applied alone or in combination with mineral fertilizers. Moreover, the lack of a consistent trend in both NDF and DNDF (47.2–54.4%) across locations highlights a clear fertilizer × environment interaction, indicating that environmental conditions exerted a stronger influence than fertilization. According to Marchesini et al. [65], the NDF content typically decreases with increasing grain proportion due to a dilution effect. However, this trend was not consistently observed in the present study, suggesting that environmental conditions may have limited the expected reduction in fiber content, regardless of fertilization treatment. Overall, the differences between this study and previous findings highlight a clear fertilizer × environment interaction, where the effect of fertilization on forage quality is dependent on site-specific climatic conditions. These results emphasize that environmental factors can override fertilization effects, particularly in determining fiber composition and digestibility.

**Table 4:** Description of the field trials conditions in Caslav, Ivanovice, and Lukavec. The verbal assessment of mean annual temperature and sum of precipitation was conducted according to [68].

	Caslav		Ivanovice na Hane		Lukavec	
GPS coordinates	49°85′ NL; 15°40′ EL		49°19′ NL; 17°05′ EL		49°34′ NL; 14°59′ EL	
Elevation (m a.s.l.)	263		225		620	
Soil type	Chernozem degraded		Chernozem		Cambisol	
MAT 2020 (°C)	10.6	Extraordinary warm	10.3	Very warm	9.5	Extraordinary warm
MASP 2020 (mm)	679	Normal	719	Wet	956	Very wet
MAT 2021 (°C)	9.4	Extraordinary warm	9.4	Normal	8.0	Normal
MASP 2021 (mm)	520	Normal	580	Normal	650	Normal
MAT 2022 (°C)	10.7	Extraordinary warm	10.3	Wery warm	9.0	Very warm
MASP 2022 (mm)	511	Normal	540	Normal	787	Wet
MAT 2023 (°C)	11.7	Extraordinary warm	10.9	Extraordinary warm	9.1	Extraordinary warm
MASP 2023 (mm)	523	Normal	667	Wet	619	Normal
Long–term MAT (1961–2019; °C)	9.1		9.0		7.5	
Long–term MASP (1961–2019; mm)	516		552		689	

Note: MAT—mean average temperature; MASP—mean average sum of precipitation.

## 4. Materials and Methods

### 4.1. Trial Areas

The trials described in this article were established at three locations with different soil–climate conditions in 1956, located in Central Europe, within a warm summer continental climate: (1) Caslav, (2) Ivanovice na Hane, (3) Lukavec. The evaluation covers a period of four seasons (2020–2023). The fundamental characteristics of the sites are presented in Table 4. The basic chemical properties of the soil prior to the assessment period (2019) are presented in Table 5.

### 4.2. Trial Description

In each area, the experiment consisted of four fields. In each field, 16 experimental plots measuring 8 × 8 m were established, and four fertilizer treatments were applied in a completely randomized block design, with four replications for each treatment. The four treatments included (1) Control (unfertilized), (2) a solid farmyard manure (FYM), (3) farmyard manure with the addition of mineral N (FYM + N), and (4) farmyard manure with the addition of mineral NPK (FYM + NPK). The FYM, sourced from cattle, was applied annually by hand in the autumn before the spring planting of maize at a rate of 40 t ha^−1^. Following the application, the FYM was incorporated into the soil by shallow plowing. The mean chemical properties of the FYM (DM; 2020–2023) were N—2.2%; P—0.6%; K—3.8%; Ca—3.3%; Mg—0.6%; organic matter—56.4%, DM—24.7%; pH—8.9. The doses of mineral N, P, and K fertilizers in the FYM + N and FYM + NPK treatments were 100, 80, and 100 kg ha^−1^, respectively. The forms of mineral fertilizers used were lime ammonium nitrate with lime (27% N), triple superphosphate (19.4% P), and potassium chloride (49.8% K). All mineral fertilizers were spread by hand on the individual plots. Each year, the preceding crop was winter wheat, and the maize hybrid utilized in the trial was a relatively new hybrid called Prestol (FAO 260, SAATEN-UNION CZ s.r.o., Šaratice, Czech Republic)), producing tall to very tall plants with excellent foliage and a dominant head, ensuring a very balanced yield/quality ratio during harvest. Crop planting was conducted depending on soil and climatic conditions from the second half of April to the first half of May, and harvesting from mid-August to mid-September (later dates correspond to the Lukavec site with cooler weather and higher altitude). Seeding rate was approximately 95 thousand per hectare with the spacing 0.7 × 0.15 m.

### 4.3. Biomass Quality Analyses

Following the harvest, together eight entire maize plants from each experimental plot were harvested, dried at 50 °C ± 5 °C under intensive ventilation for 24 h, and subsequently milled to a particle size of less than 1 mm, in accordance with CSN 467090 and EC Commission Regulation 152/2009. The cuttings were then analyzed using the FOSS NIR Systems 6500 dispersion spectrometer (NIR Systems, Inc., Silver Spring, MD, USA). Measurements were conducted in small ring cups with two parallel repetitions. Sample scanning was performed in reflectance mode over the 400–2500 nm range (covering the visible and near–infrared regions of the spectrum), with a scanning step of 2 nm. The analysis of maize silage quality—specifically crude protein (CP), fiber content (FB), neutral detergent fiber (NDF), starch content (STR), organic matter digestibility (OMD), and digestible neutral detergent fiber (DNDF)—was carried out using WinISI II software (Infrasoft International, Inc., Piscataway, NJ, USA), version 1.50, based on calibration equations developed according to the methodological procedures published by Nerušil et al. [69].

### 4.4. Soil Chemical Analyses

Following the harvest, soil samples were collected from the depths of 0.00–0.15 m (Lukavec) and 0.00–0.20 m (Ivanovice, Caslav). A total of three soil samples were collected from each plot using a stainless steel soil probe (usually Royal Eijkelkamp, Giesbeek, The Netherlands). The pH of the soil was measured after shaking for 2 h in the suspension of 0.2 M KCl. The total C and N were determined by combustion analysis using a Vario Max analyzer (Elementar Analysensysteme GmbH, Hanau, Germany). Content of plant–available forms of phosphorus (P), potassium (K), magnesium (Mg), and calcium (Ca) was analyzed by the extraction in Mehlich 3 reagent [70], followed by ICP–OES analysis (Thermo Jarrell Ash, Trace Scan, Franklin, TN, USA).

### 4.5. Data Analysis

The data were checked for normal distribution using the Shapiro–Wilk [71] and Anderson–Darling tests [72] (XLStat (Lumivero, Burlington, MA, USA)). Based on the normality test results, the data were analyzed by ANOVA, followed by Tukey’s HSD post hoc test, or by Kruskal–Wallis ANOVA, followed by Conover–Iman [73] post hoc test (Statistica 14.0 (Tibco Software, Palo Alto, CA, USA), XLStat (Lumivero, Burlington, MA, USA)). Effects of several factors (locality, treatment, season) were analyzed by MANOVA (Hoteling–Lawley’s test, XLStat (Lumivero, Burlington, MA, USA)). Relationships among data were analyzed by PCA [74] (XLStat (Lumivero, Burlington, MA, USA)). According to the analysis, the fixed variables were fertilizer treatments and localities.

## 5. Conclusions

This four-year study showed that farmyard manure (FYM) applied alone provided biomass yields comparable to FYM applied with mineral fertilizers (N, NPK), indicating that additional mineral fertilizers may not be economically and environmentally justified. While increased nutrient doses showed a trend toward higher biomass, the improvements were not statistically significant. FYM enhanced the availability of key nutrients like calcium, magnesium, and carbon, which are absent in mineral fertilizers, making it a sustainable and cost-effective option for farmers.

While application of mineral fertilizers was not connected with significantly higher BIY or silage maize quality parameters, the positive effect on soil pool nutrients was recorded, leaving the soil enriched for the following crops. The combined application of FYM + NPK significantly increased N, P, K, and C contents across all localities. The FYM, applied alone, enhanced C and N levels; however, it has limited effects on P and K unless combined with inorganic fertilizers.

## Figures and Tables

**Table 1:** The differences in biomass yield (BIY, t ha^−1^) across various trial sites, years, and fertilization treatments. Average values (±SE) that share the same letter (^a^ vertically, ^A^ horizontally) are statistically comparable (α < 0.05).

	2020	2021	2022	2023	Mean
Caslav					
Control	15.3 ± 0.6 ^A^	14.5 ± 0.4 ^A^	13.0 ± 0.6 ^A^	18.9 ± 0.2 ^B^	15.4 ± 0.6 ^A^
FYM	19.0 ± 0.5 ^B^	16.9 ± 0.3 ^B^	16.6 ± 0.2 ^B^	19.9 ± 0.2 ^C^	18.1 ± 0.4 ^B^
FYM + N	18.0 ± 0.6 ^B^	18.2 ± 0.1 ^BC^	19.2 ± 0.6 ^C^	21.5 ± 0.1 ^D^	19.2 ± 0.4 ^B^
FYM + NPK	18.8 ± 0.5 ^B^	19.6 ± 0.7 ^C^	21.8 ± 0.1 ^D^	18.0 ± 0.2 ^A^	19.6 ± 0.4 ^B^
Mean	17.8 ± 0.4 ^ab^	17.3 ± 0.5 ^a^	17.6 ± 0.9 ^ab^	19.5 ± 0.3 ^b^	
Ivanovice					
Control	21.5 ± 0.4 ^A^	18.8 ± 1.1 ^A^	25.1 ± 1.1 ^A^	16.6 ± 0.7 ^A^	20.5 ± 0.9 ^A^
FYM	21.7 ± 0.8 ^A^	17.8 ± 0.8 ^A^	36.4 ± 1.0 ^C^	19.9 ± 1.0 ^AB^	23.9 ± 1.9 ^B^
FYM + N	26.2 ± 0.6 ^B^	19.7 ± 1.3 ^A^	32.7 ± 0.3 ^B^	21.9 ± 0.6 ^B^	25.1 ± 1.3 ^B^
FYM + NPK	28.8 ± 0.9 ^B^	19.4 ± 0.5 ^A^	32.8 ± 0.5 ^B^	21.4 ± 1.0 ^B^	25.6 ± 1.4 ^B^
Mean	24.5 ± 0.9 ^b^	18.9 ± 0.5 ^a^	31.7 ± 1.1 ^c^	19.9 ± 0.7 ^a^	
Lukavec					
Control	10.2 ± 1.1 ^A^	14.8 ± 1.2 ^A^	17.0 ± 0.9 ^A^	16.7 ± 0.8 ^A^	14.7 ± 0.8 ^A^
FYM	16.9 ± 1.7 ^B^	19.5 ± 0.3 ^B^	20.8 ± 1.4 ^AB^	22.3 ± 0.7 ^AB^	19.9 ± 0.7 ^B^
FYM + N	18.9 ± 1.6 ^B^	18.5 ± 0.4 ^AB^	24.0 ± 1.3 ^BC^	26.0 ± 0.7 ^B^	21.8 ± 1.0 ^B^
FYM + NPK	18.9 ± 1.4 ^B^	21.4 ± 1.7 ^B^	26.4 ± 0.8 ^C^	22.7 ± 2.6 ^AB^	22.4 ± 1.0 ^B^
Mean	16.2 ± 1.1 ^a^	18.5 ± 0.8 ^ab^	22.1 ± 1.0 ^b^	21.9 ± 1.1 ^b^	

**Table 2:** The soil chemical composition in Caslav, Ivanovice, and Lukavec, as affected by fertilizer treatments. Average values (±SE) that share the same letter (^a^ vertically, ^A^ horizontally) are statistically comparable (α < 0.05).

	Control	FYM	FYM + N	FYM + NPK	Mean
Caslav					
pH	6.7 ± 0.1 ^AB^	6.9 ± 0.1 ^B^	6.5 ± 0.1 ^A^	6.6 ± 0.1 ^AB^	6.7 ± 0.1 ^b^
P (mg kg^−1^)	36 ± 6 ^A^	53 ± 5 ^A^	37 ± 6 ^A^	132 ± 14 ^B^	65 ± 8 ^a^
K (mg kg^−1^)	92 ± 3 ^A^	132 ± 4 ^A^	109 ± 4 ^A^	172 ± 20 ^B^	126 ± 7 ^a^
Ca (mg kg^−1^)	2964 ± 101	3441 ± 248	2912 ± 108	3104 ± 159	3105 ± 87 ^b^
Mg (mg kg^−1^)	133 ± 17	171 ± 22	155 ± 11	149 ± 13	152 ± 8 ^b^
C_tot_ (%)	1.9 ± 0.1 ^A^	2.3 ± 0.2 ^B^	2.1 ± 0.1 ^AB^	2.2 ± 0.1 ^AB^	2.1 ± 0.1 ^a^
N_t_ (%)	0.15 ± 0.01	0.16 ± 0.01	0.17 ± 0.01	0.17 ± 0.01	0.16 ± 0.01 ^a^
Ivanovice					
pH	6.7 ± 0.1	6.8 ± 0.1	6.8 ± 0.1	6.7 ± 0.1	6.8 ± 0.1 ^b^
P (mg kg^−1^)	69 ± 6 ^A^	117 ± 12 ^B^	92 ± 8 ^AB^	162 ± 14 ^C^	110 ± 8 ^b^
K (mg kg^−1^)	171 ± 10 ^A^	287 ± 22 ^BC^	249 ± 17 ^AB^	347 ± 29 ^C^	264 ± 15 ^b^
Ca (mg kg^−1^)	4179 ± 108	4190 ± 85	4231 ± 97	4052 ± 131	4163 ± 52 ^c^
Mg (mg kg^−1^)	186 ± 4 ^A^	214 ± 6 ^B^	220 ± 5 ^B^	228 ± 9 ^B^	212 ± 4 ^c^
C_tot_ (%)	2.7 ± 0.1 ^A^	3.0 ± 0.1 ^AB^	3.1 ± 0.1 ^B^	3.2 ± 0.1 ^B^	3.0 ± 0.1 ^b^
N_t_ (%)	0.19 ± 0.01 ^A^	0.22 ± 0.01 ^AB^	0.22 ± 0.01 ^AB^	0.23 ± 0.01 ^B^	0.21 ± 0.01 ^b^
Lukavec					
pH	5.7 ± 0.1	5.8 ± 0.1	5.6 ± 0.1	5.7 ± 0.1	5.7 ± 0.1 ^a^
P (mg kg^−1^)	34 ± 1 ^A^	69 ± 3 ^B^	38 ± 1 ^A^	149 ± 6 ^C^	73 ± 8 ^a^
K (mg kg^−1^)	112 ± 6 ^A^	153 ± 8 ^BC^	139 ± 10 ^AB^	176 ± 7 ^C^	145 ± 6 ^a^
Ca (mg kg^−1^)	2121 ± 77	2090 ± 80	2170 ± 69	2174 ± 89	2139 ± 38 ^a^
Mg (mg kg^−1^)	96 ± 6	101 ± 6	94 ± 6	83 ± 5	94 ± 3 ^a^
C_tot_ (%)	2.5 ± 0.1 ^A^	2.8 ± 0.1 ^AB^	3.0 ± 0.1 ^B^	3.0 ± 0.1 ^AB^	2.8 ± 0.1 ^b^
N_t_ (%)	0.21 ± 0.01	0.22 ± 0.01	0.24 ± 0.01	0.23 ± 0.01	0.23 ± 0.01 ^b^

Note: lines without a letter do not differ from one another in a statistically significant way.

**Table 3:** The impact of various fertilizer treatments and locations on the quality parameters of silage maize biomass (% of dry matter content, followed by the standard error). Summary of results for the four-year period (2020–2023).

	Control	FYM	FYM + N	FYM + NPK
Caslav				
CP (%)	7.6 ± 0.3	7.8 ± 0.3	8.0 ± 0.5	7.7 ± 0.2
FB (%)	20.9 ± 1.3	21.0 ± 0.7	21.3 ± 0.8	21.2 ± 1.7
NDF (%)	47.1 ± 2.4	47.0 ± 2.4	48.6 ± 1.0	47.8 ± 2.6
STR (%)	32.0 ± 0.2	32.3 ± 0.3	31.8 ± 0.4	30.9 ± 1.1
OMD (%)	68.9 ± 1.5	70.9 ± 1.8	68.9 ± 1.2	67.6 ± 2.8
DNDF (%)	54.4 ± 2.3	53.9 ± 1.2	52.9 ± 0.9	52.8 ± 1.3
Ivanovice				
CP (%)	7.9 ± 0.3	7.4 ± 0.3	8.0 ± 0.3	8.2 ± 0.4
FB (%)	18.6 ± 1.0	23.0 ± 2.1	17.8 ± 1.1	18.9 ± 1.6
NDF (%)	45.1 ± 1.0	51.0 ± 1.7	43.2 ± 2.0	45.9 ± 1.9
STR (%)	33.4 ± 0.7	31.0 ± 1.3	33.1 ± 0.8	33.3 ± 1.3
OMD (%)	69.8 ± 1.6	65.2 ± 1.5	69.1 ± 2.5	68.9 ± 3.3
DNDF (%)	49.4 ± 0.4	51.1 ± 2.1	47.7 ± 1.0	49.0 ± 1.2
Lukavec				
CP (%)	8.0 ± 0.1	7.7 ± 0.1	8.5 ± 0.5	8.8 ± 0.5
FB (%)	18.4 ± 0.7	18.0 ± 0.7	18.5 ± 1.7	19.0 ± 1.2
NDF (%)	43.8 ± 1.3	41.9 ± 1.7	44.5 ± 3.1	43.9 ± 2.6
STR (%)	32.5 ± 0.5	32.9 ± 0.4	32.2 ± 1.2	32.6 ± 1.0
OMD (%)	69.4 ± 3.0	67.0 ± 2.8	69.1 ± 4.1	68.1 ± 3.3
DNDF (%)	49.0 ± 0.9	49.0 ± 1.4	49.6 ± 1.5	47.2 ± 1.4

Note: CP—crude protein; FB—fiber content; NDF—neutral detergent fiber; STR—starch content; OMD—digestibility of organic matter; DNDF—digestibility of neutral detergent fiber.

**Table 5:** Characteristics of the soil at individual locations in 2019 (*n* = 4).

Characteristics	Lukavec	Čáslav	Ivanovice
pH_KCl_ (-)	5.7 ± 0.04	6.6 ± 0.2	6.4 ± 0.2
pH_H2O_ (-)	6.6 ± 0.03	7.5 ± 0.2	7.1 ± 0.2
C_t_ (%)	1.3 ± 0.05	1.0 ± 0.03	1.7 ± 0.11
N_t_ (%)	0.2 ± 0.01	0.2 ± 0.01	0.2 ± 0.01
Ca (mg kg^−1^)	1971 ± 99	3092 ± 191	3939 ± 216
K (mg kg^−1^)	116 ± 5.3	116 ± 7	182 ± 4
Mg (mg kg^−1^)	91 ± 13	125 ± 10	197 ± 26
P (mg kg^−1^)	42 ± 1.3	45 ± 16	62 ± 15

Note: analyses are described in Section 4.4.

## Data Availability

The original contributions presented in this study are included in the article/Supplementary Material. Further inquiries can be directed to the corresponding author.

## Supplementary Materials

The following supporting information can be downloaded at: https://www.mdpi.com/article/10.3390/plants15081231/s1.

## Abbreviations

The following abbreviations are used in this manuscript:

FYM	Farmyard manure
NPK	Mineral fertilizers; N—nitrogen; P—phosphorus; K—potassium
BIY	Biomass yield
CP	Crude protein
FB	Fiber content
NDF	Neutrally detergent fiber
STR	Starch content
OMD	Organic matter digestibility
DNDF	Digestibility of neutral detergent fiber
